# P-1519. A Novel Pseudomonas aeruginosa Virulence Factor, CdiA-CT, Promotes Pathogenesis via Host tRNA Cleavage

**DOI:** 10.1093/ofid/ofaf695.1703

**Published:** 2026-01-11

**Authors:** Adam S Bronson, Jonathan P Allen, Abby Kroken

**Affiliations:** Loyola University Chicago Stritch School of Medicine, Maywood, IL; Loyola University Chicago, Maywood, Illinois; Loyola University Chicago Stritch School of Medicine, Maywood, IL

## Abstract

**Background:**

*Pseudomonas aeruginosa* (*Pa*), an opportunistic and often multidrug-resistant bacterial pathogen, encodes an array of virulence factors that promote invasion and survival within the host. A recently identified *Pa* virulence factor, contact-dependent growth inhibition toxin A (CdiA-CT), is a ribonuclease that cleaves eukaryotic tRNAs, disrupts cellular homeostasis, and promotes mortality in mice during bloodstream infection.

**Methods:**

Here, we use an AI-powered structure prediction approach to predict the molecular specificity of CdiA-CT. We leveraged the AlphaFold3 platform to screen the binding of 414 human tRNA species to *Pa* CdiA-CT. To investigate how CdiA-CT's tRNAse activity impacts inflammation, we measured cytokine secretion in murine bone marrow-derived macrophages infected with WT, *ΔcdiA-CT*, and H3372A (catalytic null) mutants via cytometric bead array. Lastly, we measured bacterial fitness and host inflammation in a BALB/cJ murine bacteremia model.

**Results:**

AlphaFold3 screening revealed preferential binding to glutamine and certain valine tRNA isoacceptors, some of which contain immunostimulatory sequences known to activate TLR7, a single stranded RNA sensor. *In vitro* infections of murine bone marrow-derived macrophages with WT, *ΔcdiA-CT*, and H3372A (catalytic null) mutants showed that tRNAse activity stimulates the release of numerous proinflammatory cytokines, including IL-6, IL-10, and TNF-α. Notably, treatment of macrophages with a TLR7-specific inhibitor demonstrated that this response is largely dependent upon TLR7. Finally, we observed that despite having a stark *in vivo* attenuation, CdiA-CT deletion and catalytic mutants do not have fitness defects during bloodstream infection. We posit that this attenuation is due to less pathogenic inflammation, which is known to contribute to mortality in severe *Pa* infections.

**Conclusion:**

Overall, our results suggest that CdiA-CT is a specific tRNAse that promotes inflammation via TLR7 sensing of tRNA cleavage products, the first observation of its kind. In an age of widespread antibiotic resistance, we suggest that combining traditional antibiotics with immune modulating adjuvants may be a promising strategy for improving patient outcomes.

**Disclosures:**

All Authors: No reported disclosures

